# Targeting Tinnitus via Masseter Muscle Stimulation: Innovative Pencil‐Electrode TENS Therapy for Bruxism‐Associated Tinnitus

**DOI:** 10.1002/brb3.71443

**Published:** 2026-04-28

**Authors:** Zehra Aydoğan, Nazife Öztürk Özdeş, Sevgi Kutlu, Kübra Binay Bolat, Emre Ocak

**Affiliations:** ^1^ Audiology Department, Faculty of Health Sciences Ankara University Ankara Turkey; ^2^ Audiology and Speech Disorders Department, Institute of Health Sciences Ankara University Ankara Turkey; ^3^ Otorhinolaryngology Department, Faculty of Medicine Ankara University Ankara Turkey

**Keywords:** bruxism, qualities of life, tinnitus, transcutaneous electrical nerve stimulation

## Abstract

**Purpose:**

This study investigated the effects of pencil‐electrode transcutaneous electrical nerve stimulation (TENS) applied to the masseter muscle on tinnitus severity, bruxism‐related discomfort, and quality of life in individuals with chronic bruxism‐associated tinnitus and normal hearing.

**Methods:**

Thirty‐one adults with normal hearing thresholds (≤ 20 dB HL at 0.5–4 kHz) and chronic subjective tinnitus related to bruxism were randomly assigned to a TENS group (*n* = 18; mean age: 41.4 ± 11.8 years) or a control group (*n* = 13; mean age: 36.9 ± 8.8 years). TENS was administered four times per week for 5 weeks. Outcome measures included the Tinnitus Handicap Inventory (THI), Visual Analog Scale (VAS; 0–10, assessing bruxism‐related jaw discomfort and tinnitus severity), Generalized Anxiety Disorder‐7 (GAD‐7), Oral Behavior Checklist (OBC), mandibular range of motion (ROM), and SF‐36 Quality of Life Scale. All questionnaires were administered at baseline and after completion of treatment.

**Results:**

The TENS group demonstrated significant reductions in THI total and subscale scores, VAS bruxism‐related discomfort and tinnitus severity, anxiety (GAD‐7), and oral parafunctional behaviors (OBC), along with significant improvements in mandibular ROM and SF‐36 pain and general health subscales (*p* < 0.05). No significant changes were observed in the control group.

**Conclusion:**

Pencil‐electrode TENS applied to the masseter muscle appears to be an effective, non‐invasive treatment for bruxism‐associated tinnitus, potentially acting through somatosensory neuromodulation mechanisms.

## Graphical abstract

An overview of the study design and principal results regarding masseter‐targeted pencil‐electrode TENS in individuals suffering from bruxism‐related tinnitus. A total of 31 participants (TENS: *n* = 18; Control: *n* = 13) underwent treatment over a period of 5 weeks. The focused stimulation of the masseter, temporalis, and TMJ led to a decrease in tinnitus severity and discomfort associated with bruxism, alongside enhancements in mandibular range of motion (ROM) and overall quality of life, thereby indicating a somatosensory neuromodulation effect.

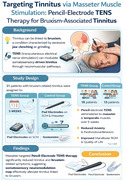

## 1 Introduction

Tinnitus is a symptom characterized by phantom auditory sensations in the absence of auditory stimuli, with a global prevalence of 10%–25% (Henry et al. 2005). In approximately 1%–3% of the general population, tinnitus is severe enough to affect quality of life, causing sleep disturbance, concentration problems, and psychiatric distress (Dobie 2003; Li et al. 2022).

Because otologic conditions are a major risk factor for tinnitus, auditory phantom sensations are often considered to be a neuroplastic response to sensory deprivation. However, this relationship is not straightforward; some individuals with tinnitus have normal hearing, while many individuals with hearing loss do not report tinnitus. Therefore, tinnitus cannot be explained solely by altered firing patterns along the tonotopic organization of a damaged cochlea. Despite extensive research, the underlying pathophysiology of tinnitus remains incompletely understood (Baguley et al. 2013).

In addition to auditory system–related factors, increasing evidence suggests that somatosensory disorders may play a significant role in the onset and modulation of tinnitus. TMD is a term used to describe a range of problems involving the temporomandibular joint (TMJ), masticatory muscles, and associated structures (McNeill 1997). The etiology of TMD is recognized as multifactorial, with bruxism recognized as one of its most prevalent contributing factors. Bruxism is classified by the American Academy of Sleep Medicine (AASM) as a stereotyped movement disorder characterized by teeth grinding and clenching, particularly during sleep (Kato et al. 2001). Excessive contraction of the masticatory muscles in bruxism may alter somatosensory input transmitted via trigeminal and cervical pathways, which converge with auditory processing centers, particularly the dorsal cochlear nucleus. This abnormal sensory integration is thought to contribute to tinnitus perception in susceptible individuals (Kato et al. 2001).

One of the most reported somatosensory conditions associated with tinnitus is temporomandibular disorder (TMD) (Bernhardt et al. 2011). The prevalence of tinnitus in TMD patients ranges from 33% to 76%, which is substantially higher than that observed in the general population (Ramírez et al. 2007). Beyond epidemiological studies, the causal relationship between tinnitus and TMD has been investigated by Ren and Isberg (Ren and Isberg 1995), who examined the prevalence of tinnitus and TMD and found that 94.3% of patients with unilateral tinnitus and unilateral anterior disc displacement had both symptoms on the same side. It has also been reported that tinnitus intensity and mandibular movements correlate with chewing or pressure on the TMJs (Ren and Isberg 1995).

Recently, transcutaneous electrical nerve stimulation (TENS) has been used as an effective treatment for tinnitus. It is believed that TENS therapy reduces the existing spontaneous nerve discharge as a result of hyperpolarization in the peripheral nerve network of the eighth nerve (at the lower basilar membrane levels of the cochlea) with positive current, and thus tinnitus regresses (Steenerson and Cronin 2003).

For bruxism, TENS is a relaxation and pain‐relieving treatment. Muscles contract and shorten when they spasm. This can cause pain and other dysfunctions by reducing blood flow and sending unwanted toxins, such as lactic acid, into the muscle. TENS helps muscles relax. TENS delivers mild, rhythmic electrical stimulation to the nerves that control facial expression and chewing muscles. The rhythmic vibration increases blood flow to the tense muscles, helping them to relax and detoxify. It may therefore be a treatment option.

While TENS has been applied to cervical and auricular regions in tinnitus management, there is a notable lack of studies examining the direct stimulation of the masseter muscle for tinnitus relief, particularly in individuals with bruxism. This study aims to address this gap by exploring the neuromuscular and somatosensory relationship between bruxism and tinnitus.

The main aim of this study was to investigate the effects of TENS on tinnitus severity, bruxism discomfort, and quality of life in people with normal hearing and chronic tinnitus caused by bruxism. In addition, both tinnitus and bruxism have become important public health problems that are prevalent in society, negatively affecting quality of life and causing financial and psychological losses. Determining the effectiveness of masseter‐targeted TENS therapy may contribute to multidisciplinary treatment strategies and offer clinicians a novel, non‐invasive perspective in the management of bruxism‐associated tinnitus.

## 2 Methods

This study included data collected between 2023 and 2024 at the Audiology Unit of the ENT Department, Faculty of Medicine, Ankara University. Informed consent was obtained from all participants before the study, and the principles of the Declaration of Helsinki were followed. Ethics committee approval was obtained from Human Research Ethics Committee, Faculty of Medicine, Ankara University, No. I02‐57‐23.

### 2.1 Study Participants

A total of 31 participants, who had been diagnosed with both bruxism and chronic subjective tinnitus, were included in this study. Participants aged between 18 and 60 years were included.

The participants were randomly assigned to a TENS treatment group and a control group. The diagnosis of bruxism was made by an ENT specialist based on a combination of clinical signs (e.g., masticatory muscle tenderness, tooth wear facets, and TMJ discomfort) and patient‐reported oral behaviors, assessed via the Oral Behavior Checklist (OBC). Chronic subjective tinnitus was defined as a self‐reported perception of tinnitus lasting for at least 6 months. Patients diagnosed with bruxism by the ENT (Ear, Nose, and Throat) and complaints about tinnitus for at least 6 months were included in the study. Patients who had not received any treatment for tinnitus and/or bruxism in the last year were included in the study. Individuals were randomly divided into two groups: a control group (*n* = 13) and a TENS treatment group (*n* = 18). Randomization was performed using an online randomization tool (randomizer.org) based on participants’ file numbers.

Inclusion criteria were normal otoscopic findings; normal hearing (pure tone thresholds < 20 dB at 0.5, 1, 2, and 4 kHz and air‐bone gap < 10 dB HL); normal tympanogram (with type A); normal limits of ipsilateral and contralateral acoustic reflex thresholds (at 0.5, 1, 2, and 4 kHz); and absence of systemic disease. Exclusion criteria were abnormal hearing and immittance measurements; hypertension, hyperlipidemia, or diabetes; any middle ear pathology such as otosclerosis or otitis media; diagnosis of acoustic neuroma (monitored or treated); previous ear and/or cranial surgery; Meniere's disease or active ear discharge; and any neuropsychiatric disease. Patients with contraindications to TENS (pacemaker, pregnancy, etc.) were also excluded.

#### 2.1.1 Methods

The pure‐tone test, acoustic immittance assessment, extended high‐frequency audiometry, and psychoacoustic evaluation of tinnitus were performed with the AC‐40 clinical audiometer (Interacoustics, Denmark). Tinnitus loudness matching (dB SL) and tinnitus pitch matching (Hz) were performed using standard psychoacoustic procedures. The Visual Analog Scale (VAS), GAD 7 (General Anxiety Disorder), OBC, mandibular range of motion (ROM), and Tinnitus Handicap Inventory (THI) were also used to measure tinnitus severity and discomfort. The Short Form 36 (SF‐36) Quality of Life Scale was used to measure the impact of tinnitus on quality of life. All questionnaires were administered at baseline and immediately after completion of the 5‐week intervention period.

Bruxism was clinically assessed by an ENT specialist based on a combination of patient‐reported symptoms (e.g., jaw pain, morning headache, awareness of clenching, or grinding), clinical signs (e.g., masseter hypertrophy, and tooth wear facets), and dental history, when available. Palpation of the masseter and temporalis muscles was also performed to detect tenderness or hypertrophy. While polysomnographic confirmation was not available, clinical criteria and standardized questionnaires (such as the OBC) were used to support the diagnosis.

#### 2.1.2 Patient Assessment Form

This form was administered by the clinician for information purposes. It consisted of questions including demographic information (age, gender, and education level); time of onset of bruxism and tinnitus; treatment method for tinnitus; hearing loss; buzzing and fullness in the ear; hyperacusis; and presence of tinnitus. The forms were completed by the clinician verbally asking the participants.

#### 2.1.3 Visual Analog Scale (VAS)

VAS assessed jaw pain, bruxism‐related discomfort, and tinnitus severity on a 0–10 scale. Tinnitus severity and pain related to TMJ dysfunction and bruxism were also assessed using the VAS. Participants were asked to rate their average jaw‐related pain or soreness during the past 4 weeks on a 0–10 scale, where 0 indicated “no pain” and 10 indicated “worst possible pain”.

#### 2.1.4 Tinnitus Handicap Inventory

The THI, one of the most widely used questionnaires, was used to determine the severity and level of tinnitus. The total THI score ranges from 0 to 100, and a higher THI score indicates greater disability related to tinnitus (Aksoy et al. 2007).

#### 2.1.5 SF‐36 Quality of Life Scale

This was used to analyze the health status and quality of life of individuals. This scale contains 36 items and assesses eight subdimensions. The score of each sub‐domain on the scale varies between 0 and 100. The higher the score of each sub‐domain, the higher the health‐related quality of life (Koçyiğit et al. 1999).

#### 2.1.6 Gad 7 (Anxiety Level Questionnaire)

Anxiety level was assessed. This questionnaire consists of seven items assessing anxious mood and behavior. The total score ranges from 0 to 21 points. The higher the score, the higher the level of anxiety (Konkan et al. 2013).

#### 2.1.7 Mandibular Range of Motion (ROM)

**FIGURE 1:**
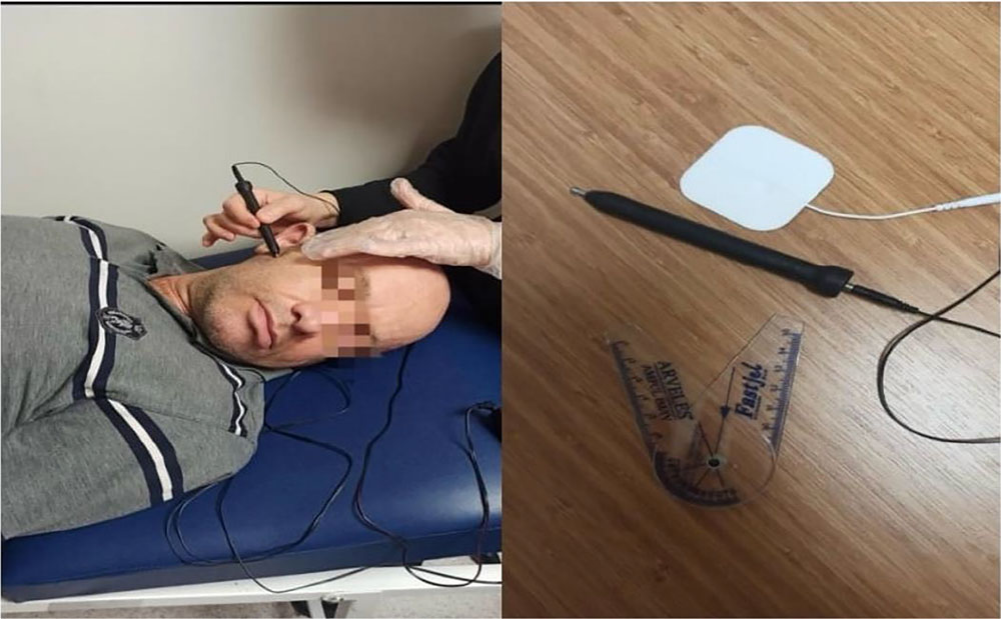
TENS application, pen electrode, and goniometer.

Mandibular ROM was assessed by maximum unaided mouth opening. Maximum mouth opening was recorded in millimeters (mm) by opening the mouth at the patient's pain limit and measuring the distance between the incisal edge of the anterior incisors and the incisal edge of the lower incisors (Figure 1).

#### 2.1.8 Oral Behavior Checklist

Oral parafunctional habits were assessed. The questionnaire consists of 21 items asking about behaviors such as holding the jaw forward or to the side, inserting the tongue between the teeth, and holding objects such as hair, nails, pens, etc. between the teeth. The total score ranges from 0 to 84 points. The higher the score, the higher the pitch of parafunctional behaviors (Schiffman et al. 2014; Kaplan and Ohrbach 2016).

#### 2.1.9 Tens

Following the assessment, 20 sessions of TENS treatment (30 min, four sessions per week over 5 weeks) were administered to individuals who met the inclusion criteria. A traditional TENS device was applied by the clinician to the masseter and temporalis muscles, as well as the TMJ and the sternocleidomastoid (SCM) region. Pad electrodes (carbon silicon component) and pencil electrodes were used for application (Figure 1). Pad electrodes were placed on the SCM muscle, parallel to the muscle fibers, to ensure stable contact and broad stimulation. Pencil electrodes were applied directly over the trigger points of the masseter and temporalis muscles and around the TMJ area, allowing for precise stimulation of these anatomically small and complex regions. Unlike conventional TENS studies, the use of pencil electrodes allowed for more targeted stimulation of anatomically small and complex areas, such as the TMJ and the muscle insertions, thereby enhancing the precision and efficacy of the intervention. The TENS parameters included a frequency of 50–100 Hz, current intensity of 40–70 mA, and voltage amplitude of 10–30 V. Each session lasted 30 min and was completed without any complications.

The selected application sites were based on the potential neuromuscular and somatosensory mechanisms underlying the relationship between bruxism and tinnitus. The masseter and temporalis muscles are primary muscles involved in bruxism; TENS is applied to these areas to reduce muscle hyperactivity, relieve pain, and improve mandibular function. The TMJ is functionally and anatomically linked to both bruxism and tinnitus, and its dysfunction may contribute to otologic symptoms. The SCM muscle, connected to cervical and mandibular biomechanics, has also been implicated in somatosensory modulation of tinnitus. Therefore, the combined stimulation of these regions was designed to address both musculoskeletal and sensory components contributing to bruxism‐related tinnitus.

### 2.2 Statistical Analysis

Data were analyzed using IBM SPSS Statistics standard software V25.0. Sample size was determined using G*Power version 3.1 software. Descriptive statistics were presented. An independent two‐sample *t*‐test was used to compare age and duration of complaints between groups. Intergroup comparisons of numerical variables with a normal distribution before and after treatment were performed by two‐way repeated measures analysis of variance. The independent *t*‐test was used. For total score values that did not show a normal distribution before and after treatment, intergroup comparisons were made using the Mann–Whitney *U* test and within‐group comparisons using the Wilcoxon test. In all analyses, *p* < 0.05 was considered statistically significant.

## 3 Results

A total of 31 individuals, including 10 females (55.55%) and eight males (44.44%) in the study group and eight females (61.63%) and five males (38.46%) in the control group, were included in our study. Demographic and descriptive data of the patients are shown in Table 1.

**TABLE 1:** General characteristics of participants (demographic information).

	Case group	Control group	*p*
Gender (male/female %)	44.4 %–55.5%	38.46%–61.53%	
Age (years)	41.38 ± 11.83	36.92 ± 8.81	0.120
Tinnitus duration (years)	4.55 ± 3.82	6.76 ± 3.78	0.172
Bruxism complaint duration	4.72 ± 3.75	6.69 ± 3.73	0.117
Tinnitus sound type (%)			
Ringing	55.5	53.8	
Breeze	16.6	15.3	
Other	27.7	23.0	
Education (%)			
Primary school	22.2	15.3	
Secondary‐high	11.1	15.3	
School university +	61.1	69.2	

*Note*: Data have presented as mean ± standard deviation or percentage.

The mean age of the study and control groups was 41.38 ± 11.83 and 36.92 ± 8.81 years, respectively, and gender, age, duration of tinnitus, duration of bruxism complaints, type of tinnitus, and educational status were reported between the groups. No statistically significant difference was found (*p* > 0.05).

There was no significant difference in the pitch of tinnitus in both groups when comparing within and between groups (Table 2). Tinnitus loudness is expressed in dB sensation level (dB SL), and tinnitus pitch is expressed in Hz. VAS scores range from 0 (no discomfort) to 10 (worst imaginable discomfort). In the study group, tinnitus loudness, VAS TMJ pain severity, VAS bruxism discomfort rating, VAS tinnitus severity, and VAS discomfort rating were significantly different before and after TENS treatment (Table 2).

**TABLE 2:** In‐group and between‐group comparison (Tinnitus pitch, tinnitus loudness, VAS [severity and annoyance]).

	Case Group a	Case Group b	*p*	95% CI	Control Group a	Control Group b	*p*	95% CI	Inter Group a/p b/p	
Tinnitus frequency (Hz)	5.44 ± 2.03	5.33 ± 1.81	0.579	[−0.30/0.52]	5.07 ± 1.75	4.92 ± 1.55	0.673	[−1.87/3.41]	0.604	0.515
Tinnitus loudness (dB SL)	44.16 ± 13.20	35.27 ± 15.47	**0.001** ^**^	[5.63/12.14]	38.46 ± 10.68	37.69 ± 10.44	0.539	[−1.87/3.41]	0.737	**0.002** ^*^
VAS (TMJ‐pain severity)	6.38 ± 1.94	4.55 ± 1.42	**0.001** ^**^	[0.47/1.30]	4.53 ± 0.96	4.61 ± 1.12	0.794	[−0.42/−0.89]	0.490	**0.006** ^*^
VAS (TMJ‐brucsizm annoyance)	6.5 ± 1.79	4.22 ± 1.16	**0.001** ^**^	[1.48/3.07]	5.00 ± 0.81	4.76 ± 0.83	0.461	[−0.55/0.89]	0.536	**0.001** ^**^
VAS (tinnitus severity)	5.83 ± 1.54	4.66 ± 1.18	**0.002** ^*^	[1.50/0.35]	4.69 ± 1.54	4.61 ± 1.12	0.784	[−0.55/−0.70]	0.710	**0.035** ^*^
VAS (tinnitus annoyance)	5.06 ± 1.63	4.64 ± 1.60	**0.025** ^*^	[1.16/0.37]	3.00 ± 1.08	3.30 ± 0.94	0.392	[0.44/−0.88]	0.070	**0.036** ^*^

*Note*: Data were presented as mean ± standard deviation and CI (confidence interval). VAS: Visial Analog Scale. TMJ:Temporomandibular joint Case Group: TENS treatment Control Group: Control. Case Group a: before TENS, Case Group b: after TENS, Control Group a: before Group b: after (8 weeks later). Within‐group changes were presented as

^*^ *p* < 0.05 and ^**^ *p*< 0.01.

Bold values indicate statistically significant differences (*p < 0.05, p < 0.01).

In the control group, there was no statistically significant difference in tinnitus loudness, VAS‐TMJ pain severity, VAS‐bruxism discomfort rating, VAS‐tinnitus severity, or VAS‐discomfort rating between the first and last evaluation (*p* > 0.05) (Table 2). While there was no significant difference between the groups at the first visit (*p* > 0.05), there was a significant difference at the last visit for tinnitus severity, VAS‐TMJ pain severity, VAS‐bruxism discomfort, VAS‐tinnitus severity, and VAS‐discomfort. A decrease and an improvement in VAS scores were observed in the study group (Table 2).

When quality of life was assessed before and after TENS treatment, a significant difference was found in the sub‐parameters of SF‐36 in EP, E, P, and GH in the study group (*p* < 0.05) (Table 3). In the control group, no significant difference was found in the sub‐parameters of the SF‐36 (Table 3). Between the groups, no significant difference was found at the first evaluation (*p* > 0.05), whereas a significant difference was found at the last evaluation in the sub‐parameters of SF‐36 in P and GH (*p* < 0.05) (Table 3).

**TABLE 3:** In‐group and between‐group comparison (SF‐36).

Sf‐36	Case group (min/max, mean ± SD)				Control group (min/max, mean ± SD)				Intergroup	
	Case Group a	Case Group b	*p*	95% CI (lower/upper)	Control Group a	Control Group b	*p*	95% CI (lower/upper)	a, *p*	b, *p*
PF	71.94 ± 18.64	75.00 ± 16.17	0.102	[−6.78/0.67]	72.69 ± 15.62	77.30 ± 12.51	0.116	[−13.29/4.93]	0.907	0.671
PR	49.16 ± 22.96	55.27 ± 21.79	0.127	[−14.13/1.91]	53.84 ± 22.96	48.97 ± 21.55	0.864	[−5.18/16.30]	0.617	0.369
EP	55.16 ± 30.20	62.50 ± 25.28	**0.042**	[−14.34/−0.31]	63.69 ± 25.42	66.07 ± 13.83	0.431	[−15.32/10.55]	0.415	0.647
E/F	68.33 ± 8.40	50.66 ± 25.27	0.017	[−9.00/−2.26]	70.92 ± 6.50	72.30 ± 6.65	0.140	[−5.71/2.96]	0.363	0.686
E	71.11 ± 14.74	73.55 ± 15.83	**0.05**	[0.12/−2.01]	68.61 ± 10.56	68.61 ± 9.91	0.056	[−2.79/2.78]	0.607	0.330
SF	70.13 ± 23.45 70(25–100)	95.83 ± 13.25 95 (0–100)	0.422	[−4.17/9.52]	92.88 ± 15.70 90(25–100)	93.78 ± 5.98 90(25–100)	0.709	[−5.37/13.31]	0.894	0.364
P	57.19 ± 13.27	70.69 ± 12.88	**<0.001**	[−5.89/−3.76]	59.80 ± 13.71	59.61 ± 13.10	0.299	[−2.50/−4.94]	0.598	**0.026**
GH	68.33 ± 14.98	75.61 ± 13.76	**0.05**	[0.12/−2.10]	70.76 ± 10.19	71.46 ± 11.96	0.125	[−2.70/6.68]	0.130	**0.05**

*Note*: Data were presented as minimum, maximum, mean ± standard deviation and CI (confidence interval). The SF‐36 consists of 36 items—that are grouped in eight subscales or domains. Within‐group changes were presented as ^*^ *p* < 0.05 and ^**^ *p* < 0.01. Case Group: TENS treatment Control Group: no TENS treatment Case Group a: before TENS treatment, Case Group b: after TENS treatment, Control Group a: before, Control Group b: after.

Abbreviations: E/P, energy/fatigue; E, emotional well‐being; EP, role limitations due to emotional problems; GH, general health; P, pain; PF, physical functioning; PR, role limitations due to physical health; SF, social functioning.

Bold values indicate statistically significant differences (*p < 0.05, p < 0.01).

According to the results of the THI questionnaire, which we used to assess the severity of tinnitus; THI‐emotional (THI‐e) (*p* = 0.08), THI‐functional (THI‐f) (*p* = 0.02), THI‐catastrophic (THI‐c) (*p* = 0.01), and THI‐total score (*p* = 0.01), the decrease in scores before and after TENS treatment was significant in the study group (Table 4). In the control group, there was no significant difference in THI scores (*p* > 0.05). Significant differences were found between the groups for THI‐c (*p* = 0.01), THI‐f (*p* = 0.043), and THI‐total score (*p* = 0.011) (Table 4).

**TABLE 4:** In‐group and between‐group comparison (THI).

THI	Case Group a	Case Group b	*p*	95% CI (lower/upper)	Control Group a	Control Group b	*p*	95% CI (lower/upper)	a, *p*	b, *p*
THI/E	11.77 ± 6.89	9.38 ± 6.66	**0.008**	[0.78/0.72]	14.46 ± 9.93	14.93 ± 9.37	0.987	[−0.69/0.69]	0.382	0.087
THI/C	11.11 ± 3.44	7.55 ± 4.63	**<0.001**	[0.67/2.12]	12.66 ± 3.35	13.07 ± 2.76	0.058	[−5.82/−0.94]	0.262	**0.001**
THI/F	15.33 ± 7.91	9.22 ± 6.96	**0.002**	[3.68/8.53]	13.30 ± 3.81	12.92 ± 2.39	0.072	[−5.50/0.27]	0.077	**0.043**
THI total	38.11 ± 14.72	24.44 ± 13.59	**<0.001**	[9.61/17.72]	37.38 ± 13.40	37.53 ± 11.67	0.781	[−2.05/2.66]	0.889	**0.011**

*Note*: Data were presented as mean ± standard deviation and CI (confidence interval). THI: Tinnitus Handicap Inventory consists of 24 items—that are grouped in three subscales or domains; THI/E: THI Emotional, THI/C: THI Catastrophic, THI/F: THI Functional. Case Group1: TENS treatment Control Group: no TENS treatment Case Group a: before TENS treatment, Case Group b: after TENS treatment, Control Group a: before, Control Group b: after.

Bold values indicate statistically significant differences (*p < 0.05, p < 0.01).

When comparing the results of the GAD‐7 questionnaire used to measure anxiety levels, the results in the study group were statistically significant compared to the post‐treatment results. The post‐treatment anxiety level in the study group was significantly lower (*p* = 0.01), whereas there was no difference between the baseline and final scores in the control group (*p* > 0.05). When the GAD‐7 scores were compared between groups, there was no difference in the baseline scores (*p* > 0.05).

When comparing the final scores of the study and control groups, there was a statistically significant decrease in GAD‐7 scores after treatment (*p* = 0.01).

When comparing the OBC scores, it was observed that the OBC scores decreased and improved statistically significantly in the study group after treatment (*p* = 0.03), while there was no difference in the control group (*p* > 0.05).

When comparing the study and control groups, while there was no difference in the initial assessment scores (*p* > 0.05), when comparing the final assessment scores, there was a statistically significant decrease and improvement in OBC scores after treatment (*p* = 0.05).

When comparing the pre‐ and post‐treatment ROM scores in the study group, a statistically significant difference was found. The post‐treatment ROM was significantly higher in the study group (*p* = 0.01), whereas there was no difference between the initial and final assessments in the control group (*p* > 0.05). When comparing the ROM measurements between the groups, there was no difference in the initial assessment scores (*p* > 0.05), while a significant difference was observed in the final assessment scores (*p* = 0.002). In‐group and between‐group comparisons for GAD‐7, ROM, and OBC are given in Table 5.

**TABLE 5:** In‐group and between‐group comparison (GAD‐7, Oral Behaviors Checklist, Mandibular Range of Motion).

Case group					Control group				Intergroup	
	Case Group a	Case Group b	*p*	95% CI (lower/upper)	Control Group a	Control Group b	*p*	95% CI (lower/upper)	a, *p*	b, *p*
GAD‐7	10.11 ± 3.80	5.66 ± 2.32	**0.001**	[3.35/5.85]	9.38 ± 2.78	9.84 ± 3.05	0.786	[−1.35/1.05]	0.739	**0.001**
OBC	25.05 ± 7.74	21.50 ± 6.71	**0.003**	[1.01/1.41]	26.23 ± 6.84	25.69 ± 6.78	0.428	[−0.89/1.96]	0.665	**0.05**
ROM (mm)	34.11 ± 3.54	40.05 ± 4.45	**0.001**	[1.47/6.94]	35.38 ± 2.54	35.84 ± 2.11	0.854	[−1.27/1.72]	0.265	**0.002**

*Note*: Data were presented as mean ± standard deviation and CI (confidence interval). Case Group1: TENS treatment Control Group: no TENS treatment Case Group a: before TENS treatment, Case Group b: after TENS treatment, Control Group a: before, Control Group b: after.

Abbreviations: GAD‐7, General Anxiety Disorder; mm, millimeter; OBC, Oral Behaviors Checklist; ROM, mandibular range of motion.

Bold values indicate statistically significant differences (*p < 0.05, p < 0.01).

## 4 Discussion

Since the 1800s, TENS has been used to treat not only pain but also muscle relaxation, inflammation, edema, ligament dysfunction, and spinal disorders (Lee et al. 2014). TENS is also thought to modulate tinnitus symptoms by increasing blood flow to the cochlea and capturing abnormal sensory signals. Electrical stimulation for the treatment of tinnitus remains an attractive therapeutic option and is used as an alternative method (Lee et al. 2014).

TENS also reduces TMJ pain in bruxism patients, relaxes contracted muscles, creates an environment for stretching shortened tissues, and is therefore a highly effective method of treatment. Bruxism is one of the most damaging parafunctional activities for the stomatognathic system (Manfredini et al. 2013; Shetty et al. 2010 Sep), so a significant proportion of parafunctional studies have focused on bruxism (Manfredini et al. 2013). It is the cause not only of oral symptoms such as tooth wear and pain, but also of symptoms such as sensitivity due to myofascial trigger points in the masticatory muscles, postural problems, depression, reduced sleep quality, attention problems, loss of motivation, restriction of mandibular jaw movements, headaches, dizziness and another common symptom, tinnitus (Amorim et al. 2018). Based on all this, we hypothesized that TENS would be an effective and attractive alternative method for the treatment of tinnitus combined with bruxism. In contrast to earlier research that utilized TENS on the cervical or auricular areas for tinnitus treatment, our investigation is pioneering in examining the impact of direct stimulation of the masseter muscle, potentially indicating a more targeted neuromuscular modulation pathway in tinnitus associated with bruxism.

Previous studies have demonstrated the potential efficacy of TENS in individuals with chronic tinnitus. In a randomized study involving 65 patients, 62.2% of those receiving TENS twice weekly for 4 weeks reported improvement compared to placebo. Similarly, another study with 46 participants found significant reductions in tinnitus loudness, THI, and VAS scores following a 20‐session TENS protocol, despite no change in tinnitus pitch (Lee et al. 2014; Aydoğan et al. 2022).

Our study included tinnitus sufferers with bruxism. The study group received TENS treatment for 5 weeks, 4 days a week for 30 min. TENS is a treatment for both tinnitus and bruxism. TENS has also been tried in the treatment of bruxism, and it has been found that it can play an effective role in relieving pain in the masticatory muscles, relaxing the jaw muscles, and increasing the opening of the TMJ (Amorim et al. 2018). For this treatment, the pencil electrode was applied to the TMJ muscles, masseter, trapezius, and SCM muscles, while the other pad electrode was applied to the upper trapezius muscle at level C2. Tinnitus pitch and loudness, THI total and subscores, VAS tinnitus severity and discomfort, VAS TMJ pain, and bruxism discomfort were compared after treatment. There was a decrease in bruxism discomfort. However, there were also improvements in tinnitus loudness and VAS severity and discomfort. This tells us that bruxism may be effective in reducing the severity of tinnitus and that bruxism treatment may also be effective for existing tinnitus complaints.

The symptom of bruxism negatively affects quality of life by causing pain and functional limitations in individuals. Since its etiology is multifactorial, the goals of bruxism treatment, in which multidisciplinary approaches have been found to increase treatment success, can be listed as reducing pain, resolving functional limitations by increasing joint mobility, and improving quality of life by providing postural correction. Similarly, tinnitus negatively affects quality of life and becomes a public health problem (Aydoğan et al. 2022). In one study, 22 patients with tinnitus were treated with TENS. SF‐36 scores, which are used to assess quality of life, were compared before and after TENS. Statistically significant improvements were observed in the scores for fatigue, social isolation, and emotional problems and in many of the parameters measured by the SF‐36, and it was emphasized that TENS is a useful method to improve the quality of life of tinnitus patients (Aydemir et al. 2006).

In our study, we used the SF‐36 questionnaire to assess quality of life. According to the results of the questionnaire, improvements were observed in the SF‐36 E/P, E, P, and GH subscores of the study group before and after TENS treatment. We have shown that TENS is an effective method, especially for pain.

Various physical and neuromodulator interventions have shown positive effects on anxiety and symptoms associated with individuals with bruxism and tinnitus. In a randomized trial, both massage and relaxation therapies led to significant improvements in anxiety, stress, and sleep quality among bruxism patients. Similarly, in individuals with chronic tinnitus, TENS applied to the ear significantly reduced THI and DASS scores compared to sham treatment. Consistent with these findings, our study also demonstrated a significant reduction in anxiety levels as measured by the GAD‐7 after TENS treatment (Miotto et al. 2021; Tutar et al. 2020).

Previous research has shown that physiotherapy interventions, including TENS, can lead to improvements in mouth opening, jaw function, and pain levels among individuals with TMDs. In studies comparing active and sham TENS, significant increases in mandibular ROM and reductions in pain were consistently reported (Moger et al. 2011; Zhang et al. 2020). These findings align with our results, which demonstrated improved mouth opening (ROM) after TENS in individuals with bruxism‐related symptoms.

Similarly, in our study an increase, a significant improvement, was observed when mouth opening ROM was measured before and after treatment. In individuals with bruxism, limitation of mouth opening is common due to muscle pain and insufficient muscle activity. We believe that the decrease in muscle pain, muscle relaxation, and ligament relaxation after TENS treatment may be the reason for the increase in ROM. Thus, we have demonstrated the positive effects of TENS on both bruxism and tinnitus. The beneficial effects of TENS observed in the present study may be related to somatosensory modulation mechanisms. Clinically, the effectiveness of TENS in bruxism‐associated tinnitus may be explained by its ability to reduce excessive masseter muscle contraction and normalize abnormal sensory input transmitted via trigeminal and cervical pathways. By modulating these inputs, TENS may decrease aberrant neural activity contributing to tinnitus perception.

The therapeutic effect of TENS on tinnitus in patients with bruxism may be related to the modulation of somatosensory input from the masticatory and cervical muscles to the auditory pathways. The trigeminal and cervical nerves, which are activated during TENS application, have direct and indirect projections to the dorsal cochlear nucleus, a structure known to integrate somatosensory and auditory signals. This neuromodulation may reduce central excitability and influence the maladaptive neural plasticity associated with tinnitus perception. Additionally, by alleviating musculoskeletal tension and improving local circulation, TENS may also contribute to the reduction of somatosensory‐driven tinnitus symptoms (Moger et al. 2011; Zhang et al. 2020). Recent systematic reviews conducted in 2025 have reinforced this connection, illustrating a bidirectional relationship in which the prevalence of tinnitus is notably elevated among individuals with TMDs (de La Torre Canales et al. 2025). Additionally, contemporary clinical evidence indicates that treatments aimed specifically at bruxism and temporomandibular dysfunction may result in significant decreases in the severity of tinnitus, thereby endorsing the somatosensory modulation hypothesis (Bousema et al. 2025). Unlike invasive or device‐dependent neuromodulation approaches such as vagus nerve stimulation or bimodal auditory‐somatosensory stimulation, masseter‐targeted TENS offers a simple, low‐cost, and clinically accessible intervention (de La Torre Canales et al. 2025; Bousema et al. 2025).

This study has some limitations. The reason for this is that the number of individuals receiving TENS treatment was not higher, and its long‐term effects were not examined. One of the other limitations of the present study is the absence of a sham‐TENS control group, which may have introduced a placebo effect. Additionally, the diagnosis of bruxism was based on clinical criteria and self‐reported behaviors rather than polysomnography, which is considered the gold standard.

## 5 Conclusion

An effective classification system based on the underlying pathophysiological mechanisms of tinnitus symptoms would be a groundbreaking step toward personalized rehabilitation and treatment protocols. We believe that the evaluation of tinnitus should be carried out by a multidisciplinary team. In the treatment of tinnitus combined with bruxism in our study, TENS treatment/therapy was found to be effective on tinnitus loudness and severity. This study suggests that TENS may have a beneficial effect on tinnitus symptoms in individuals diagnosed with bruxism. The improvements observed point toward a potential somatosensory modulation mechanism. However, the generalizability of these findings is limited, and further research into larger sample sizes and comprehensive outcome measures is needed. TENS appears to be a potentially accessible and non‐invasive adjunctive treatment modality, which may offer clinicians new perspectives in the management of tinnitus.

## Author Contributions

**Zehra Aydoğan**: conceptualization, methodology, supervision, resources, project administration, writing – review and editing, writing – original draft, investigation. **Kübra Binay bolat**: conceptualization, methodology, investigation. **Nazife Öztürk özdeş**: conceptualization, methodology, investigation, resources, supervision, writing – review and editing, writing – original draft. **Sevgi Kutlu**: conceptualization, methodology, investigation. **Emre Ocak**: conceptualization, methodology, writing – review and editing.

## Ethics Statement

Ethics committee approval was obtained from Human Research Ethics Committee, Faculty of Medicine, Ankara University, No: I02‐57‐23. Written consent forms were obtained from the participant whose photograph appears in the figure and all other participants.

## Funding

The authors have nothing to report.

## Conflicts of Interest

The authors declare no conflicts of interest.

## Data Availability

Data will be forwarded if researchers reach the corresponding author.
